# Myokine–adipokine cross-talk: potential mechanisms for the association between plasma irisin and adipokines and cardiometabolic risk factors in Mexican children with obesity and the metabolic syndrome

**DOI:** 10.1186/s13098-019-0458-2

**Published:** 2019-08-05

**Authors:** Adrian M. Gonzalez-Gil, Mariana Peschard-Franco, Elena C. Castillo, Gustavo Gutierrez-DelBosque, Victor Treviño, Christian Silva-Platas, Luisa Perez-Villarreal, Gerardo Garcia-Rivas, Leticia Elizondo-Montemayor

**Affiliations:** 1 Escuela de Medicina y Ciencias de la Salud, Tecnologico de Monterrey, Ave. Morones Prieto 3000, 64710 Monterrey, N.L. Mexico; 2 Center for Research in Clinical Nutrition and Obesity, Tecnologico de Monterrey, Ave. Morones Prieto 300, 64710 Monterrey, N.L. Mexico; 3 Cardiovascular and Metabolomics Research Group, Hospital Zambrano Hellion, Tecnologico de Monterrey, 66278 San Pedro Garza Garcia, P.C. Mexico

**Keywords:** Irisin, Obesity, Metabolic syndrome, Adipokines, Children, Pediatric, Insulin resistance, Cardiovascular disease, Inflammatory cytokines, Leptin

## Abstract

**Background:**

Adipokines and the myokine irisin, involved in mechanisms associated with obesity and metabolic syndrome (MS), are understudied in the pediatric population.

**Objective:**

To investigate the relationship between irisin, and leptin, resistin, adiponectin, adipsin, anthropometric and cardiovascular risk factors in Mexican children.

**Methods:**

A cross-sample of 126 Mexican children aged 6–12 years old were classified as normal weight (n = 46), obese (n = 40), and MS (n = 40) according to CDC’s and Cook’s age-modified criteria for obesity and MS. Anthropometric parameters and blood pressure were determined and percentiles calculated for age and gender. Irisin, leptin, adiponectin, adipsin, resistin, triglycerides, glucose, high-density lipoprotein cholesterol (HDL-c) levels, and physical activity were determined. Statistical tests for differences between groups, correlation, and multiple regression analyses were performed.

**Results:**

Irisin plasma levels were significantly lower in the obese (6.08 [4.68–6.65]) and MS groups (6.46 [5.74–7.02]) compared with the normal-weight group (8.05 [7.24–8.94]) (p < 0.001). Irisin levels were not influenced by age or gender, but significant dispersion was observed in obese girls (95% CI median [2.29–6.30]). Leptin, resistin, and adipsin levels were significantly increased in the obese and MS groups. Lean-fat ratio was significantly higher in the NW group. Irisin correlated negatively with leptin (− 0.310), resistin (− 0.389), adipsin (− 0.362), BMI% (-0.472), WC% (− 0.453), BMI z-score (− 0.496), fat free mass (− 0.257), fat percentage (− 0.532), fat mass (− 0.515), triglycerides (− 0.291), the number of cardiometabolic risk factors (− 0.443) (p < 0.001); positively with lean-fat ratio (0.489) and HDL-c (0.328) (p < 0.001) and none with physical activity (*p *< 0.001). Following stepwise multiple linear regression analysis, the lean-fat ratio was the only determinant of irisin levels (B = 1.168, p < 0.001).

**Conclusions:**

Lean-fat ratio, more than the absolute amount of muscle or fat mass, as well as potential myokine–adipokine cross-talk mechanisms may explain the lower irisin levels in children with obesity and MS, through blunted compensatory responses interfering with tissue-dependent irisin secretion, contributing to a continuous deleterious effect cycle.

**Electronic supplementary material:**

The online version of this article (10.1186/s13098-019-0458-2) contains supplementary material, which is available to authorized users.

## Background

The increasing incidence rates and prevalence of pediatric of obesity and the metabolic syndrome (MS) are alarming. According to the World Health Organization (WHO), the worldwide combined overweight and obesity prevalence in children and adolescents has increased from 4% in 1975 to 18% in 2016 [1]. Since 2016, Mexico has become the country with the highest childhood obesity rate in the world [2], with a combined overweight and obesity prevalence of 33.2% among school-age children [3]. The prevalence of the MS in the pediatric population, which has also been increasing, was found to be of 6.4% in the US according to NCEP-ATP III age-modified criteria [4]. In Mexican children, it was reported to be of 23.3% using the same criteria, ranging from 11.0% in the overweight group to 73.9% in the severely obese group [5].

The secretion of molecules by muscle and adipose tissue, namely myokines and adipokines, particularly that of irisin, has recently become a focus of research in the field of pediatric obesity [6], as they might play an important pathophysiologic role in metabolic dysfunction and its complications [7, 8]. The positive correlations between leptin levels and anthropometric and metabolic parameters in children with obesity and the MS are well known [9–11]. Overwhelming evidence indicates that hyperleptinemia and leptin resistance have a central pathogenic role in the blunted anorexigenic effect and suppressed energy expenditure [12, 13], as well as in the systemic proinflammatory state observed in obesity and in the MS [7, 14]. Resistin, another adipokine, has been shown to induce inflammation [15], endothelial dysfunction, and atherogenesis [16]. The evidence regarding the role of resistin in obesity and MS in children has been shown to be conflicting. Some authors have reported positive correlations between resistin and markers of obesity and the MS in children [17–19], while others have not [20, 21]. Adipsin, another adipokine, has been attributed proadipogenic properties by triggering triglyceride synthesis and adipocyte differentiation [22]. To our knowledge, the role of adipsin in children with obesity or the MS has not been characterized to date. Finally, adiponectin has been found to be negatively correlated with fat mass [23], to improve insulin sensitivity, and to exert anti-inflammatory and anti-atherosclerotic effects [24, 25]. The vast majority of studies in children have demonstrated a negative correlation between adiponectin and markers of obesity [9, 26] and the MS [21, 27].

Irisin, a recently discovered myokine/adipokine, is a 112 amino acid cleavage product of fibronectin type III domain-containing protein 5 (FDNC5), which is in turn stimulated by peroxisome proliferator-activated receptor-γ co-activator 1α (PGC-1α). Originally described in mice by Böstrom et al., irisin was shown to increase the expression of uncoupling protein-1 (UCP-1) and to stimulate the “browning” of subcutaneous white adipose tissue, resulting in increased energy expenditure, improvement of insulin resistance, and possibly weight loss [28]. Although irisin was first identified as a myokine secreted by muscle in response to exercise, it was later identified to be also secreted by white adipose tissue [29], hence the term adipomyokine. Irisin has been recently implicated in obesity and the MS as a potential regulatory molecule involved in both insulin resistance and weight reduction in adults [30]. Most clinical studies in the adult population agree on a positive correlation between irisin concentrations and body mass index (BMI) [31–34]. The same results have been described for the association of irisin with parameters of the MS. Most of the studies in adults have also found a positive association between irisin concentration and cardiometabolic risk factors of the MS [35–38], while a few have shown an inverse relationship [39] or none at all [40, 41]. On the contrary, correlation of irisin levels in the pediatric population with obesity remains a topic of controversy [42]. While some authors have found irisin to be positively correlated with BMI percentile, waist circumference (WC) and fat-free mass [43–46], others have found a negative correlation [47] or none at all [48, 49]. Furthermore, fewer studies have been conducted regarding the relationship between plasma irisin levels and cardiometabolic risk factors of the MS in children, with even more inconsistent results. Some have reported positive correlations between irisin and some cardiometabolic risk factors of the MS [45, 50], while a negative correlation [47] or no association [48] have also been shown.

Irisin could be linked to other regulatory hormones such as adipokines, an association which may intertwine in the maintenance of metabolic status [51]. Adipokines such as leptin and resistin have been found to be consistently increased in both adults and children with obesity and the MS, contributing to insulin resistance. Basic and clinical studies in adults have found diverse associations between irisin and adipokines. A positive association between irisin and leptin [52] and adiponectin [53] has been described, while a negative relationship of irisin with leptin [54, 55], adiponectin [35, 56, 57], and resistin [58, 59], or no correlation with any of them have also been found [48, 60, 61]. Noteworthy, studies associating irisin concentrations with the different adipokines in the pediatric population are both limited and contradictory [42], and to date, no data has been found for adipsin. A positive correlation between irisin and leptin [43, 45] or no correlation at all [46] has been described. A single study in a cohort of children has reported no correlation between irisin and resistin [48]. A few in vitro and in animal studies have described the interplay between leptin and irisin. Administration of leptin in mice has been found to increase FNDC5 expression in skeletal muscle, but to decrease FNDC5 expression in subcutaneous adipose tissue (SAT) by downregulating PGC-1α. Co-treatment of subcutaneous adipocytes with leptin and irisin has proved to diminish irisin-induced fat browning [52]. Further insight into newly discovered molecules, such as irisin, in the context of childhood obesity and the MS is needed. As well, the interplay between irisin and adipokines is still scarcely described in the pediatric population. Thus, the objective of this study was to characterize the association between irisin and adipokines, as well as with cardiometabolic risk factors and anthropometric parameters in children with obesity and the MS. In addition, the interplays of the potential myokine–adipokine cross-talk as mechanisms to explain these associations and the metabolic and inflammatory implications is emphasized.

## Methods

### Population

A cross-sample of 126 Mexican children (65 girls and 61 boys) aged 6–12 years old was included. Children were classified into two groups according to the BMI percentile based on the criteria established by the Centers for Disease Control and Prevention (CDC) [62]. Children with a BMI ≥ 5th and < 85th percentiles (25 girls and 21 boys) according to age and sex were categorized as normal weight (NW) and those with a BMI ≥ 95th percentile (40 girls and 40 boys) according to age and sex as obese (OB). Additionally, children with obesity were further classified according to the presence (20 girls and 20 boys) or absence (20 boys and 20 girls) of the MS, as defined by Cook et al. [63]. Children in the OB and MS subgroups were matched by BMI percentile. Inclusion criteria included attendance to elementary school (first to sixth grade), Mexican ethnicity, and a 12-h overnight fast. The exclusion criterion was disapproval by the children’s primary care physician because of any medical conditions that could limit their participation in the study. Approval was obtained from the Ethics and Research Committees of the School of Medicine of Tecnologico de Monterrey (13CI19039138). All parents or legal guardians gave their written informed consent. The participants did not receive any form of compensation for participating in this study.

### Definition of metabolic syndrome and cardiometabolic risk factors

The MS was defined according to Cook, et al. [63], as the presence of ≥ 3 of the following cardiometabolic risk factors: (1) WC ≥ 90th percentile for age and gender; (2) fasting glucose ≥ 100 mg/dL; (3) systolic (SBP) or diastolic blood pressure (DBP) ≥ 90th percentile for age, gender, and height; (4) High density lipoprotein cholesterol (HDL-c) ≤ 40 mg/dL; or (5) TG ≥ 110 mg/dL. Fasting glucose was modified from ≥ 110 mg/dL to ≥ 100 mg/dL in order to match the American Diabetes Association’s definition of impaired fasting glucose [64].

### Blood pressure and anthropometric parameters

Anthropometric variables and blood pressure were measured by qualified physicians. Systolic and diastolic blood pressure measurements were performed in triplicate using a mercury sphygmomanometer with an appropriate cuff size, with the patient seated in appropriate position, waiting 1 min between each blood pressure reading. Normal values for SBP and DBP in the pediatric population correspond to measures with values < 90th percentile for age and gender; values > 90th percentile and < 95th percentile are considered as prehypertension and values > 95th percentile are considered as hypertension according to the National Heart Lung and Blood Institute (NHLBI) guidelines [65]. Anthropometric measurements were obtained in accordance with standardized protocols [66]. Weight in kilograms (kg), rounded to the nearest decimal point, and fat percentage (%) were obtained with an age-appropriate scale (TANITA ^®^ BF-689; TANITA Corporation of America Inc, Arlington Heights, Illinois, USA). Muscle mass and fat free mass were attained by formula. Mid-upper arm circumference (MUAC) (cm) was measured with a flexible fiberglass tape around the mid-upper arm at the midpoint between the olecranon and the acromion. The mid-upper arm muscle circumference (MUAMC) (cm) was determined as MUAC − [(3.14159) × (TSF mm/10)] and the mid-upper arm muscle area (MUAMA) (cm^2^) as [(MUAMC cm^2^)/(4 × 3.14159)] − 10. Muscle mass was calculated as (height-cm)[0.264 + (0.0029 × MUAMA-cm^2^], while fat-free mass was obtained as [weight-kg − (weight-kg × body fat%)] [67]. Height in centimeters (cm) was obtained with a stadiometer (SECA^®^ 217, SECA Mexico, Mexico City, Mexico), while WC (cm) was determined using a standard fiber optic measuring tape. BMI was calculated as the quotient of mass (kg)/height^2^ (m). BMI z-score was calculated based on the criteria established by the World Health Organization (WHO) [68]. The lean-fat ratio was calculated as the quotient of muscle mass (kg) and fat mass (kg).

### Physical activity

Information regarding children’s physical activity was obtained through a previously validated questionnaire filled by each child and parent/caregiver in a face-to-face interview. Information about the children’s days per week and hours per day of regular exercise was provided [69]. Questions regarding regular physical activity during the last 6 months included: (1) Whether the children exercised or not; (2) If the answer was positive, the type of exercise they engaged in, for example, aerobic (football, basketball, swimming, dancing, running, walking, cycling, among others) or anaerobic (sprinting, climbing, isometrics, or any rapid burst of hard exercise); (3) The number of days they practiced physical activity per week; and (4) The number of hours per day that they exercised. The number of hours per week was obtained multiplying the hours per day by the days per week of physical activity. All children reported aerobic exercise type and none reported anaerobic physical activity.

### Laboratory studies

Blood samples were collected from subjects by peripheral venipuncture after an overnight 12-h fast. Samples were then centrifuged, and serum and plasma were frozen at − 80 °C for further processing. Fasting serum glucose concentrations were obtained through Hexokinase/Glucose 6-Phosphate methodology using the kit Glucose 3L82 (catalog number 304772/R02; DENKA SEIKEN CO., LTD. Tokyo Japan) in the Architects cSystems™. TG were measured in plasma through the glycerol-phosphate-oxidase reaction, running the Triglyceride 7D74-20 reagent kit (catalog number 30-3140/R3; Abbot Laboratories Diagnostic Division, IL USA) in the Architect cSystems™ and the AEROSET system. High-density lipoprotein cholesterol (HDL-c) levels were quantified through the accelerator selective detergent method with the Ultra HDL 3K33-21 assay (catalog number 306571/R03; Abbot Laboratories Diagnostic Division, IL USA). Irisin levels were obtained by sandwich enzyme-linked immunosorbent assay (ELISA) using an irisin (human) ELISA kit (catalog number SK00170-08; Avisera Bioscience Inc, Santa Clara, California, USA) following the manufacturer’s instructions. The sensitivity of the assay is reported as 0.1 ng/mL with a standard curve linear range of 0.8–51.2 ng/mL. The inter- and intra -assay variation were 8–10% and 4–6% respectively. The adipokine profile was obtained from serum samples using a Human Metabolic Panel 1 (4-plex) LEGENDplex™ Multi-Analyte Flow Assay kit (BioLegend^®^, San Diego, CA, USA, catalogue number 740212). The panel included adipsin, leptin, resistin, and adiponectin. The LEGENDplex™ assays consists of bead-based immunoassays, which were performed as indicated by the manufacturer’s instructions using the FACS-Canto-II flow cytometer (BD Biosciences, San Jose, California, USA).

### Statistical analysis

Statistical analyses were performed using Microsoft Excel^®^ (version 16.17, Microsoft Corporation, Redmond, Washington, USA), IBM SPSS^®^ (version 21.0; SPSS Inc., Armonk, New York, USA) and GraphPad Prism (version 6.0, GraphPad Software, La Jolla California, USA). D’Agostino-Pearson tests were carried out for each measured variable to assess normality of the sample’s distribution. Chi square test was employed to evaluate differences in distribution of categorical variables among the groups. To compare anthropometric and metabolic variables between the three groups, Kruskall-Wallis with post hoc Dunn’s multiple comparisons test and one-way ANOVA with Holm-Sidak’s correction for multiple comparisons were performed for non-parametric and parametric data, respectively, unless otherwise specified. Spearman’s correlation coefficients were determined between concentrations of irisin, adipokines, and all biochemical and clinical parameters. Stepwise multiple linear regression was conducted as indicated. Outliers were identified using the ROUT method and removed from all analyses. A *p* value of < 0.05 was considered statistically significant for all analyses.

## Results

### Demographic, cardiometabolic, and anthropometric parameters

Table 1 shows the demographic characteristics, anthropometric and cardiometabolic parameters of the groups of children. Each group had an equal distribution of male and female participants across the age ranges described. BMI, muscle mass, fat free mass, fat mass, BMI%, WC%, fat%, and BMI-z score were significantly higher in the obese and MS groups compared with the control group. On the other hand, the lean-fat ration was significantly lower in the obese 0.433 [0.380–0.627] and the MS group 0.447 [0.345–.610] compared with the normal weight group 1.68 [1.25–2.01]. DBP % was significantly greater in the group with the MS (53.5 [41.5–69.8]) compared with the obese group (39.5 [29.3–49.8]). Glucose level was significantly higher in the MS group (84.2 ± 11.0) compared with the normal weight group (77.4 ± 11.7). TG levels were significantly higher in the MS group (170.0 [129.0–224.0]), followed by the obese (95.5 [78.5–122.0]) and the normal weight groups (81.2 [66.8–93.2]). HDL-c levels were significantly lower in children with the MS (33.5 [31.0–36.0]) followed by the obese (41.0 [35.5–47.0]) and normal weight children (45.5 [42.5–47.3]).

**Table 1:** Demographic, anthropometric, clinical, and metabolic parameters of the pediatric population

Parameter	NW (n = 46)	Ob (n = 40)	MS (n = 40)
Male (%)	21 (45.7)	20 (50)	20 (50)
Female (%)	25 (54.3)	20 (50)	20 (50)
Age (years)	8.7 ± 1.7	8.7 ± 1.5	9.1 ± 1.9
BMI	15.45 [14.8–16.3]^(B, C)^	24.3[23.3–26.5]^(A)^	26.75[24.1–29.8]^(A)^
BMI%	30.0 [25.0–50.0]^(B, C)^	98.6 [97.3–99.0]^(A)^	98.9 [97.8–99.4]^(A)^
BMI z-score	− 0.39[(− 0.6) to (− 0.1)]^(B,C)^	2.93 [2.6–3.4]^(A)^	3.06 [2.7–3.6]^(A)^
WC%	25.0 [13.8–50.0]^(B, C)^	92.0 [87.0–98.0]^(A)^	97.0 [91.3–99.8]^(A)^
Muscle mass (kg)	6.43 [5.4–7.9]^(C)^	6.56 [5.8–7.5]^(C)^	7.58 [6.5–9.0]^(A, B)^
Fat-free mass (kg)	23.05 [20.3–26.8]^(B, C)^	27.22 [23.9–31.4]^(A)^	29.06 [24.4–32.8]^(A)^
Fat %	14.77 ± 3.4^(B, C)^	33.08 ± 5.7^(A, C)^	38.46 ± 9.4^(A, B)^
Fat mass (kg)	4.17 ± 1.7^(B, C)^	13.95 ± 4.5^(A)^	19.21 ± 9.1^(A)^
Lean-fat ratio	1.68 [1.25–2.01]^(B,C)^	0.433 [0.380–0.627]^(A)^	0.447 [0.345–0.610]^(A)^
Physical activity (days per week)	1.5 [0–5]	1 [0–5]	1[0–3]
Physical activity (hours per day)	1 [0–1]	1 [0–1]	1 [0–1]
Physical activity (hours per week)	1 [0–15]	1 [0–10]	1 [0–10]
SBP%	*	68.5 [50.5–80.0]	76.5 [58.3–91.0]
DBP%	*	39.5 [29.3–49.8]**	53.5 [41.5–69.8]**
Fasting glucose (mg/dL)	77.4 ± 11.7^(C)^	80.4 ± 10.7	84.2 ± 11.0^(A)^
TG (mg/dL)	81.2 [66.8–93.2]^(B, C)^	95.5 [78.5–122.0]^(A, C)^	170.0 [129.0–224.0]^(A, B)^
HDL-c (mg/dL)	45.5 [42.5–47.3]^(B, C)^	41.0 [35.5–47.0]^(A, C)^	33.5 [31.0–36.0]^(A, B)^
Irisin (ng/mL)	8.05 [7.24–8.94]^(B, C)^	6.08 [4.68–6.65]^(A)^	6.46 [5.74–7.02]^(A)^
Leptin (pg/mL)	0.32 [0.18–0.66]^(B, C)^	0.86 [0.303–2.34]^(A)^	1.32 [0.42–6.27]^(A)^
Adiponectin (pg/mL)	10.3 [4.86–14.2]	14.9 [7.06–24.9]	14.2 [6.88–22.6]
Resistin (pg/mL)	0.0 [0.0–0.0]^(B, C)^	1.75 [1.03–4.88]^(A)^	2.45 [1.32–7.57]^(A)^
Adipsin (pg/mL)	0.64 [0.38–0.80]^(B, C)^	0.90 [0.64–1.57]^(A)^	1.20 [0.79–1.56]^(A)^

Data is expressed as median and interquartile range for variables with non-parametric distribution and mean and standard deviation for variables with parametric distribution, unless specified otherwise

Percentiles of anthropometric variables were calculated according to age and gender

BMI%, body mass index percentile for age and gender; DBP%, diastolic blood pressure percentile for age, gender, and height; HDL-c, high-density lipoprotein cholesterol; MS, metabolic syndrome; NW, normal weight; Ob, obese; SBP%, systolic blood pressure percentile for age, gender, and height; TG, triglycerides; WC%, waist circumference percentile for age and gender

* BP measurements for the control group were not obtained

** Indicates *p *< 0.05 using the Mann–Whitney test

*p *< 0.05 versus ^(A)^Normal Weight ^(B)^Obese ^(C)^Metabolic Syndrome

### Irisin plasma levels by groups, age, and gender

**Fig. 1:**
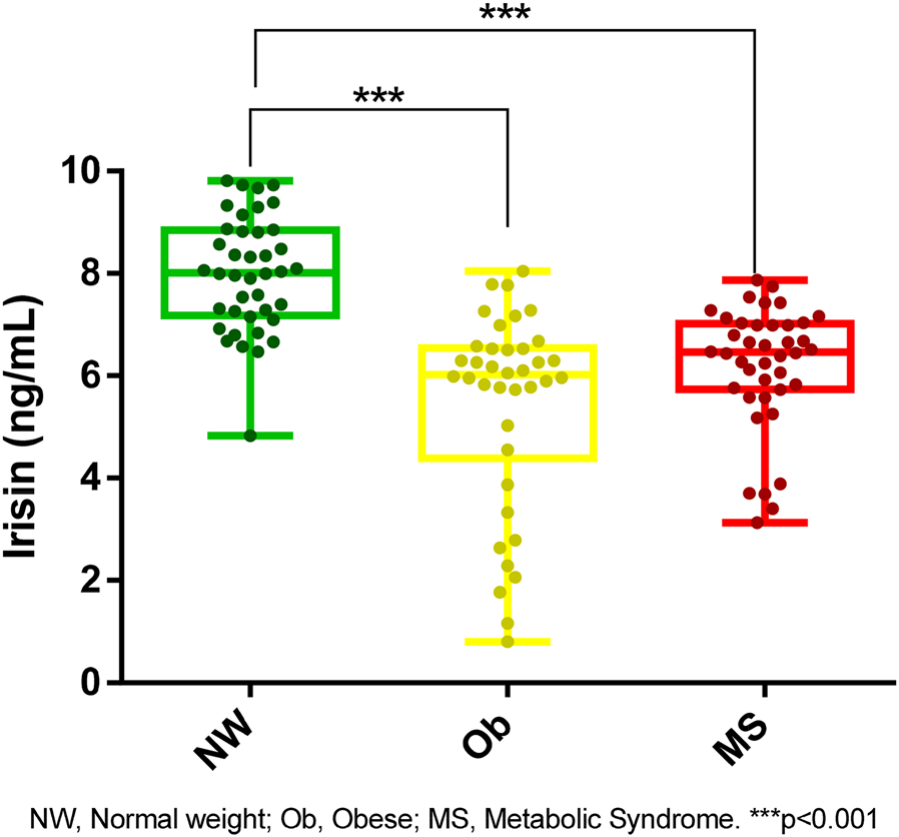
Irisin plasma levels in children with obesity, metabolic syndrome and normal weight. All data points are included; boxes represent the median and interquartile range

**Fig. 2:**
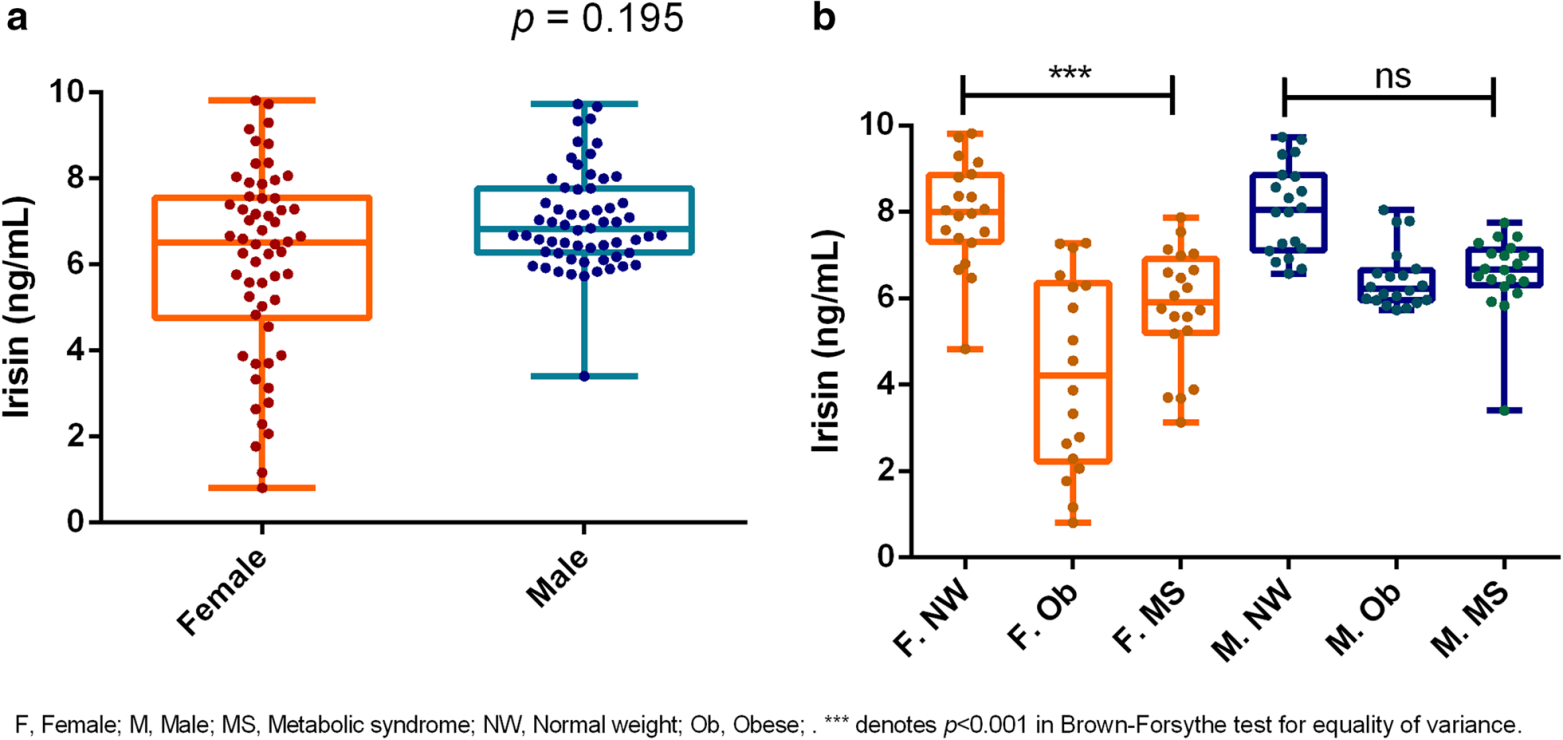
**a** Irisin levels by gender; **b** irisin levels by groups and gender. All data points are included; boxes represent the median and interquartile range

**Fig. 3:**
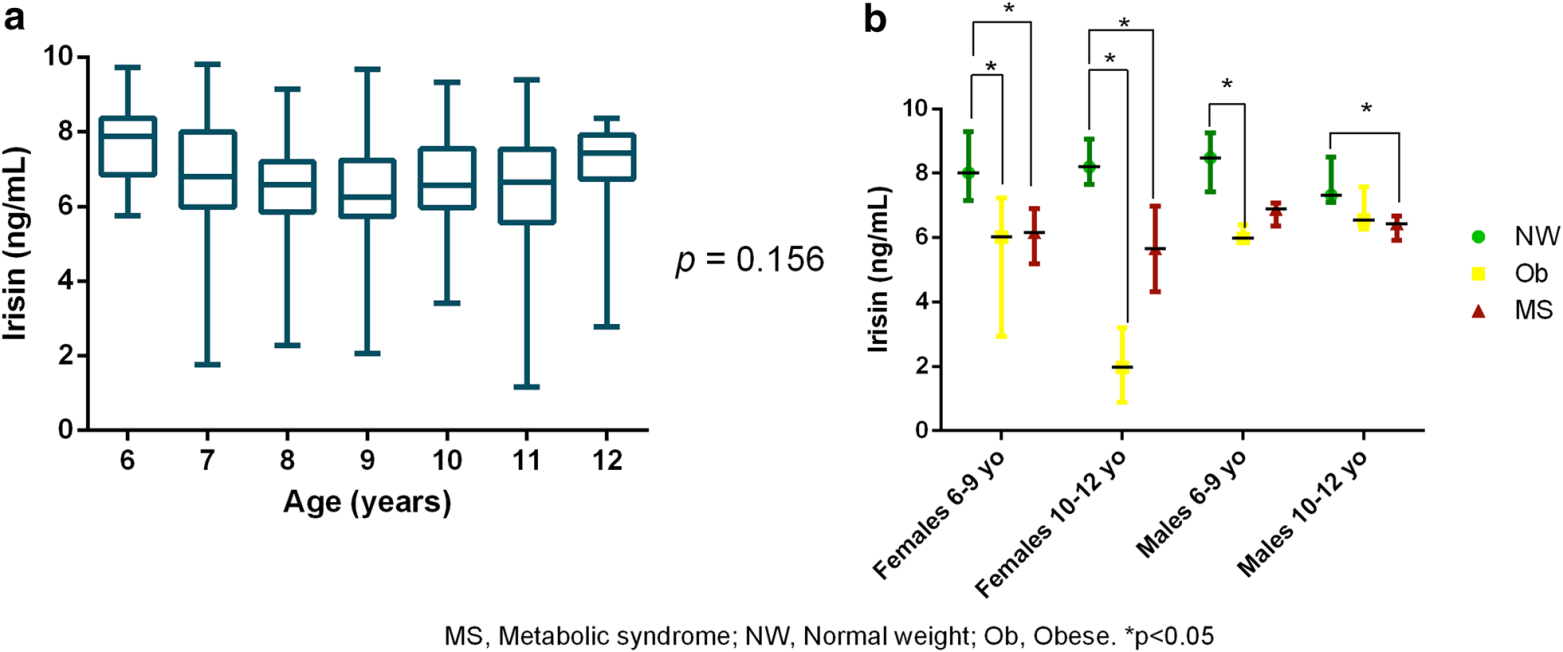
**a** Irisin levels according age; **b** irisin levels according to age, group and gender. Data presented in median and interquartile range

As shown in Fig. 1, irisin levels were significantly higher in normal weight children (8.05 [7.24–8.94]) compared with children with obesity (6.08 [4.68–6.65]) and those with the MS (6.46 [5.74–7.02]) (p < 0.001). No significant difference was observed between the obese and the MS groups. As shown in Fig. 2a, although there were no gender differences in irisin plasma values (*p* = 0.195), the dispersion of irisin concentration was significantly larger for girls compared with boys (F-test, *p* < 0.001). The irisin level dispersion was significantly different among the groups of girls (*p* < 0.001), with the greatest dispersion observed in girls with obesity (95% CI of median [2.29–6.30]), compared with girls with the MS (95% CI of median [5.26–6.65]) and normal weight girls (95% CI of median [7.39–8.80]) (Fig. 2b). No significant difference was found in the irisin levels dispersion among the three groups of boys. (p = 0.136). Age did not appear to have any influence on irisin levels when considering the total population (p = 0.156) (Fig. 3a). When divided by groups, age, and gender, irisin levels were significantly lower in girls aged 10–2 years in the obese and in the MS groups compared with the normal weight girls of the same age, although no difference was observed between the obese and the MS groups. This same pattern was also observed for girls aged 6–9 years. This trend was less evident in boys (Fig. 3b).

### Adipokines plasma levels

Leptin levels were significantly higher in the MS group (1.32 [0.42–6.27]) and the obese group (0.86 [0.303–2.34]) compared with the normal weight children (0.32 [0.18–0.66]) children, although no significant difference was observed between the obese and the MS groups. As well, resistin levels were significantly higher in the MS group (2.45 [1.32–7.57]) and the obese group (1.75 [1.03–4.88]) compared with the control group (0.0 [0.0–0.0]), but no significant difference between the obese children and those with the MS was found. A similar behavior was identified for adipsin levels which were significantly higher in the MS group (1.20 [0.79–1.56]) compared with the obese (0.90 [0.64–1.57]) and the normal weight groups (0.64 [0.38–0.80]). No significant difference was found among the three groups for adiponectin levels (Table 1).

### Correlations between irisin, adipokines, cardiometabolic, and anthropometric parameters

Diverse correlations were found between irisin plasma levels and adipokines, cardiometabolic risk factors, and anthropometric parameters. As shown in Table 2, negative correlations between plasma irisin concentration and anthropometric markers such as BMI (r_s_ = − 0.52; p < 0.001), BMI% (r_s_ = − 0.472; p < 0.001), BMI z-score (r_s_ = − 0.496; p < 0.001), fat free mass (r_s_ = − 0.257; p < 0.01), fat percentage (r_s_ = − 0.532; p < 0.001), fat mass (r_s_ = − 0.515; p < 0.001) and WC% (r_s_ = − 0.453; p < 0.001) were found. Noteworthy, lean-fat ratio was found to have a positive correlation with irisin (0.489; p < 0.001). On the other hand, a negative but weak correlation was observed between irisin and TG levels (r_s_ = − 0.291, p < 0.01) and the number of cardiometabolic risk factors (r_s_ = − 0.443; p < 0.001). HDL-c level was the only cardiometabolic risk factor shown to have a positive correlation with irisin plasma levels (r_s_ = 0.328; p < 0.001). Table 3 shows the correlation between irisin plasma levels and adipokines. Interestingly, irisin plasma levels were found to be negatively correlated with leptin (r_s_ = − 0.31; p < 0.001), resistin (r_s_ = − 0.389; p < 0.001), and adipsin (r_s_ = − 0.362; p < 0.001), as shown in Table 4. Leptin was found to be positively correlated with BMI (r_s_ = 0.46), BMI% (r_s_ = 0.329), BMI z-score (r_s_ = 0.434), WC% (r_s_ = 0.369), FFM (r_s_ = 0.329), fat % (r_s_ = 0.464), FM (r_s_ = 0.488), with the number of cardiometabolic risk factors (r_s_ = 0.341) (*p *< 0.001), and with TG levels (r_s_ = 0.254; p < 0.01), while a negative correlation with lean-fat ratio (r_s_ = − 0.376) and with HDL-c levels (r_s_ = − 0.263) (p < 0.01) was found. Similar significant results were observed for resistin and adipsin. Both resistin and adipsin levels were shown to be positively correlated with BMI, BMI%, BMI z-score, WC%, Fat %, FM, TG, and the number of cardiometabolic risk factors, and negatively associated with HDL-c levels. A weak positive correlation of adiponectin with BMI (r_s_ = 0.098), BMI% (r_s_ = 0.291), BMI z-score (r_s_ = 0.434) and WC % (r_s_ = 0.333) (p < 0.001) was found.

**Table 2:** Spearman correlation coefficients between irisin plasma levels and cardiometabolic and anthropometric parameters

	BMI	%BMI	BMI z-score	WC%	MM	FFM	Fat%	FM	LF ratio	PAdpw	PAhpd	Pahpw	SBP%	DBP%	Glc	TG	HDL-C	#RF
Irisin	− 0.520***	− 0.472***	− 0.496***	− 0.453***	*− 0.163*	− 0.257**	− 0.532***	− 0.515***	0.489***	0.105	0.124	0.045	0.013	0.19	*− *0.068	− 0.291**	0.328***	− 0.443***
BMI		0.874***	0.866***	0.790***	0.316***	0.649***	0.884***	0.933***	− 0.850***	− 0.227*	− 0.23*	− 0.202*	0.15***	− 0.061***	0.263**	0.556***	− 0.586***	*− *0.736***
BMI%			0.986***	0.906***	0.19*	0.381***	0.844***	0.815***	− 0.706***	*− 0.157*	*− 0.169*	− 0.158*	0.022	*− *0.087	0.123	0.486***	− 0.455***	0.742***
BMI z-score				0.889***	0.187*	0.398***	0.831***	0.812***	− 0.799***	*− *0.148	*− *0.165	*− *0.132	0.045***	− 0.108***	0.142	0.498***	− 0.463***	− 0.727***
WC%					0.212*	0.349***	0.797***	0.773***	− 0.747***	*− *0.105	*− *0.102	*− *0.072	*− *0.079	*− *0.021	0.153	0.474***	− 0.451***	0.783***
MM						0.537***	0.223*	0.329***	0.049	0.117	0.121	0.154	0.196	0.197	0.151	0.222*	− 0.247**	0.261*
FFM							0.442***	0.636***	− 0.465***	*− *0.082	*− *0.065	*− *0.026	0.107***	− 0.177***	0.405***	0.25**	− 0.344***	0.386***
Fat%								0.958***	− 0.916***	− 0.198*	*− 0.147*	*− 0.136*	0.043***	0.032***	0.142	0.569***	*− *0.498	0.747***
FM									− 0.902***	− 0.209*	*− 0.174*	*− 0.144*	0.078***	− 0.019***	0.233**	0.561***	− 0.532***	0.753***
LF ratio										0.277**	0.228*	0.226**	*− *0.009	0.108	− 0.199*	− 0.498***	0.461***	− 0.685***
PAdpw											0.853***	0.973***	*− *0.152	0.075	*− *0.035	*− *0.177	0.222	*− *0.136
PAhpd												0.928***	*− *0.157	0.059	*− *0.017	*− *0.086	0.167	*− *0.091
PAhpw													*− *0.14	0.091	0.002	*− *0.142	0.177*	*− *0.114
SBP%														0.415***	0.108	0.201	*− *0.051	0.307**
DBP%															0.014	0.249*	*− *0.161	0.346**
Glc																0.161	− 0.232*	0.23*
TG																	− 0.544***	0.725***
HDL-c																		− 0.708***

BMI, body mass index; BMI%, BMI percentile for age and gender; DBP%, diastolic blood pressure percentile for age and gender; Glc, glucose; HDL-c, high density lipoprotein cholesterol; LF ratio, lean-fat ratio; MM, muscle mass; FFM, fat-free mass; Fat%, body fat percentage; FM, fat-mass; PAdpw, physical activity in days per week; PAhpd, physical activity in hours per day; PAhpw, physical activity in hours per week. SBP%, systolic blood pressure percentile for age and gender; #RF, number of cardiometabolic risk factors; TG, triglycerides; WC%, waist circumference percentile for age and gender

* p < 0.05, ** p < 0.01, *** p < 0.001

**Table 3:** Spearman correlation coefficients between adipokines and cardiometabolic and anthropometric parameters

Adipokine	BMI%	WC%	LM	FM	Fat%	FFM	LF ratio	SBP%	DBP%	Glc	TG	HDL-c	#RF
Leptin	0.329***	0.369***	*0.158*	0.450**	0.443**	0.241**	− 0.376**	*− *0.061	*− *0.099	0.030	0.254**	− 0.263**	0.341***
Resistin	0.426***	0.452***	*0.047*	0.235**	0.226**	0.061	*− *0.191	*− *0.092	*− *0.011	0.055	0.330***	− 0.278**	0.411***
Adipsin	0.473***	0.481***	0.248**	0.338**	0.362**	0.097	− 0.233**	*− *0.004	0.005	*− *0.004	0.253**	− 0.314***	0.418***
Adiponectin	0.291***	0.333***	0.113	0.099	0.151	*− *0.115	*− *0.042	*− *0.098	*− *0.062	*− *0.105	0.105	*− *0.132	0.227

BMI%, BMI percentile for age and gender; DBP%, diastolic blood pressure percentile for age and gender; Fat%, percent body fat; FFM, fat-free mass; FM, fat mass; HDL-c, high density lipoprotein cholesterol; LF ratio, lean-fat ratio; LM, lean mass; SBP%, systolic blood pressure percentile for age and gender; #RF, number of cardiometabolic risk factors; TG, triglycerides; WC%, waist circumference percentile for age and gender

* p < 0.05, ** p < 0.01, *** p < 0.001

**Table 4:** Correlation between irisin concentration and adipokine levels

Adipokine	Spearman’s rho	*p*-value
Leptin	− 0.310	*< 0.001*
Resistin	− 0.389	*< 0.001*
Adipsin	− 0.362	*< 0.001*

### Physical activity

As shown in Tables 1 and 2, no correlations between physical activity and irisin, leptin, resistin or adiponectin were found. Physical activity measured in days per week was negatively correlated with BMI (r_s_ = − 0.227; p < 0.05), fat % (r_s_ = − 0.198; p < 0.05), and FM (r_s_ = − 0.209; p < 0.05), while a positive correlation between hours per week of physical activity and HDL-c (r_s_ = 0.177; p < 0.05) was found. However, these associations were weak.

Stepwise multiple linear regression analysis was conducted to determine whether body composition (lean-fat ratio), metabolic parameters (TG, HDL, and glucose levels), and physical activity in hours per week influenced irisin levels. Lean-fat ratio was found to be the only significant determinant of irisin levels with the model explaining 22.7% of the variance in levels of irisin (Irisin = 5.480 + 1.168[Lean-fat ratio]; 95% CI for B (0.779–1.558), R^2^ = 0.227, *p *< 0.001). Of importance, multiple comparison analysis revealed that the lean-fat ratio did not significantly differ between analog subgroups (see Additional file 1: Table S1). Thus, the lean-fat ratio might be used as a decisive component of irisin levels regardless of age and gender.

## Discussion

Irisin has been found to be associated with insulin resistance, obesity, the MS, cardiovascular risk factors and other metabolic diseases in adults [30]. However, data on the role of irisin as a metabolic regulator, as well as the influence of gender and age, and especially its association with adipokines in obesity and the MS in the pediatric population is still scarce and contradictory [42]. The present study provides further evidence of the association between irisin and adipokines, cardiometabolic risk factors and anthropometric parameters, elucidating a potential myokine–adipocytokine cross-talk.

### Irisin levels in relation to cardiometabolic risk factors and anthropometric markers in obesity and the MS

Our results demonstrated significantly lower plasma irisin levels in children with obesity and with the MS compared with normal weight children. Likewise, negative correlations between irisin and adiposity markers, including BMI, BMI%, BMI z-score, FFM, Fat %, and WC% levels were found. Since contradictory data in children prevails, our results diverge with some studies that have found irisin to be higher in obese children [43, 45, 46, 49, 70]. The higher levels of irisin in obesity have been explained by a compensatory increase in irisin attributed to irisin resistance or to the relative abundance of adipose tissue inducing irisin secretion. However, in agreement with our results, others have found lower irisin levels, as well as a significant negative correlation between irisin and BMI and WC in obese children compared with normal weight children [47]. These findings could be attributed to the uncertainty of whether circulating irisin originates mostly from muscle or from adipose tissue in the context of obesity [42, 54, 71, 72]. Although a direct correlation between muscle mass or fat mass and irisin levels was not observed in our study, the lean-fat ratio was significantly lower in the obese and the MS group compared with the normal weight group. In addition, the lean-fat ratio was found to be the main factor affecting irisin levels. While children with obesity and MS in our cohort were found to present significantly more muscle mass and fat mass than normal weight children, lower irisin levels were shown. Thus, we hypothesize that levels of irisin are more dependent on the proportion of muscle mass to fat mass rather than on the total quantity of either of the tissues, the reason why children with obesity and with MS might have lower irisin levels. The proportion of fat mass versus muscle mass in normal weight, overweight, obese and even underweight children has been suggested to contribute to irisin levels in another study [73]. The amount of muscle or fat mass may also be related to the underlying deleterious functional metabolic state of muscle and adipose tissue intrinsic to the context of obesity and MS. Indeed, the lean(muscle)-fat ratio has been identified as a potential indicator of future metabolic risk in the pediatric population [74], predictor of insulin resistance in patients with treatment-naïve type 2 diabetes mellitus [75], and has been proposed as a screening tool for MS in young adults [76].

Scarce and opposing evidence has also been described with reference to the role of irisin in the pediatric population with the MS [42]. Our results demonstrate an inverse correlation between irisin concentrations and TG, as well as with the number of cardiovascular risk factors of the MS. These findings are in line with those observed by Shim et al. who established inverse associations between irisin and glucose and TG levels in children [47]. In adult populations as well, irisin has been negatively associated with parameters of the MS [39] and lower levels have been correlated with higher odds of presenting an unfavorable lipid profile [77], suggesting that decreased irisin could be related to the pathway of insulin resistance and subsequent development of the MS. However, other studies have reported irisin to be positively associated with blood pressure, lipids and glucose markers, suggesting that an interaction between irisin and lipids and glucose could contribute to β-cell dysfunction and the MS [45, 50]. These inconsistent findings might be attributed to methodological differences, to ethnicity, or to body composition variances. Discrepancies in irisin levels might also be attributed to the ELISA kit used, mainly due to the sensitivity, inter- and intra-assay variability, the detection range, and the detected form of irisin in these assays [78]. Given that our results showed irisin plasma levels to have a stronger correlation with anthropometric parameters than with metabolic markers, we hypothesize that in children, irisin levels are greater influenced by the relative contribution of each body tissue type.

### Irisin levels in relation to gender and age

Our results exhibited no significant differences in plasmatic irisin levels between genders, although higher irisin plasma level dispersion was found in girls compared with boys, especially in the obese group compared with the normal weight group, which was not observed in male children. Our results are in agreement with the results from two studies with similar populations involving obese and overweight children [43, 48]. In contrast, higher levels of irisin have been found in girls compared with boys [31, 79] and in girls from the normal weight group compared with their counterpart males, but not in overweight or obese subjects [45]. Although still contradictory, the higher dispersion in irisin levels observed in the obese group of girls could be explained by higher adipose tissue mass in growing girls compared with boys or to the effects of estradiol on fat mass. With respect to the influence of age on irisin concentrations, irisin levels were not significantly affected by age in our study when considering all subjects, and when divided by age groups and weight status, similar results were found. Discrepancy about the influence of puberty on age and irisin levels has been found. An increase in irisin levels in five obese subjects entering puberty, associated with insulin resistance, has been reported [49]. In contrast, cross-sectional and interventional studies have found no association between irisin and pubertal development [45, 48, 80]. Irisin levels in our population appeared to be rather influenced by weight and obesity than by gender or age.

### Association of irisin levels with circulating adipokines

Clinical studies that explore the relationship between irisin and adipokines are scarce in the pediatric population. Our findings demonstrate higher leptin levels in the obese and MS groups compared with the normal weight group, in accordance with hyperleptinemia, which been shown to be strongly associated with fat mass in obese patients [6]. Our results also revealed a negative correlation between irisin and leptin levels. The scarce data in the pediatric population has shown a positive correlation between irisin and leptin [43, 45] or no correlation at all [46]. We hypothesize that the low levels of irisin observed in our obese and MS groups may be explained by a myokine–adipokine cross-talk that plays a modulatory role in skeletal muscle and adipose tissue cellular processes [51]. A few differing in vitro and animal studies exemplify this possibility. For instance, in mice, administration of leptin has been found to increase FNDC5 expression in skeletal muscle, but to decrease FNDC5 expression in SAT by downregulating PGC-1α. Interestingly, co-treatment of subcutaneous adipocytes with leptin and irisin proved to diminish irisin-induced fat browning [52]. These results suggest that excess leptin levels observed in obesity may interfere with the ability of irisin to promote higher energy expenditure, regardless of how much irisin is being produced. On the contrary, in another study in which rats were treated with leptin, FNDC5 expression was found to be decreased in both skeletal muscle and visceral adipose tissue (VAT), with a concurrent decrease in irisin levels, while FNDC5 expression in the hypothalamus was found to be increased. The authors proposed that leptin may have a tissue-specific role in the regulation of FNDC5 expression and the subsequent release of irisin, which may then influence global energy expenditure, through both central and peripheral mechanisms [81]. Other authors, though, have found no changes in FNDC5 expression or in circulating irisin levels after leptin treatment in mice [60, 82]. Studies with human-derived tissues have found a negative correlation between FNDC5 and leptin expression in SAT of adult subjects [54]. Leptin has been found to downregulate FNDC5 expression in subcutaneous adipocytes from non-obese adults in vitro, and positive associations between FNDC5 expression in SAT/VAT and leptin have been demonstrated in morbidly obese subjects [55]. The authors proposed that this apparent paradoxical finding could be explained by the leptin resistance condition in obesity, in which leptin may partially lose its ability to downregulate FNDC5. Overall, leptin, basally elevated in the obese state [7, 13], could regulate FNDC5 expression in a tissue-dependent manner, selectively decreasing the expression of FNDC5 in SAT, consequently decreasing circulating irisin levels. These explanations could at least partially explain the higher leptin levels and lower circulating irisin in the obese and MS groups compared with healthy controls, as well as the negative association between irisin and leptin levels found in our study. Alternatively, or concurrently, the reduced irisin levels might be attributed to impairment of FNDC5 cleavage into irisin in the context of obesity [82] or to mechanisms directly related to elevated proinflammatory cytokines as previously proposed in pediatric subjects with type 2 diabetes mellitus (T2DM) [83].

Our results also showed adipsin levels to be significantly higher in the obese and MS groups compared with the control children, as well as a negative correlation between irisin and adipsin levels. Adipsin has recently been implicated in the regulation of β-cell function. Adipsin knock-out mice were shown to develop glucose intolerance due to decreased insulin production, while administration of adipsin in diabetic mice resulted in a boost in insulin secretion and consequent decreased glucose levels, through signaling pathways downstream of C3a receptors in β-cells. In the clinical study, T2DM patients who required insulin were shown to have decreased expression of adipsin in VAT and SAT compared with insulin-naïve T2DM patients [84] Instead, in another cohort of obese adults without T2DM adipsin was found to be upregulated [85]. Therefore, adipsin could represent a protective mechanism against progression to β-cell failure and glucose levels alterations. To the best of our knowledge, the role of adipsin in childhood obesity and diabetes has not been described. Considering that none of the recruited children in our cohort presented with T2DM, we hypothesize that the higher levels of adipsin observed in the obese and MS groups compared with the normal weight group might indicate a compensatory mechanism to increase glucose tolerance in the context of childhood obesity and to possibly delay the onset of T2DM. Finally, our results also demonstrated increased resistin levels in obese children and in those with MS compared with control children, which is expected in obesity, as well as a negative correlation between irisin and resistin. A single study in a cohort of children has reported no correlation between irisin and resistin [48], while in obese men, a tendency toward decreased resistin levels along with higher levels of irisin has been described [58]. Similarly, in an animal model, rats exposed to exercise exhibited increased PGC-1α and irisin but decreased resistin expression [59]. Resistin has been shown to inhibit the translocation of GLUT-4 to the cell membranes, resulting in impaired insulin-mediated glucose uptake [86]. Conversely, irisin has been found to have the opposite effect of inducing GLUT-4 expression [87] and translocation [88]. Altogether, this explanation is in accordance with our findings in which increased resistin and decreased irisin levels in obese children and in those with MS may both compromise glucose uptake in peripheral tissues, thereby aggravating a metabolic condition.

### Irisin and physical activity

Our results showed no significant correlation between irisin and physical activity. This relationship has been studied in several transversal and interventional studies with inconsistent results. While some authors have found positive correlations between irisin and physical activity [45, 48], others have found negative associations [43, 73]. Furthermore, some evidence suggests that irisin can increase after acute bouts of exercise [80], but these elevations may not be sustained or present in long-term exercise [80, 89]. In line with our findings, other authors have also failed to find associations between irisin and exercise in certain study groups [80, 90]. The heterogeneity of the studies, combined dietetic interventions, different types and duration of exercise, the tools used to measure physical activity, as well as body composition, make it difficult to interpret the influence of exercise over levels of irisin.

### Cross-talk between irisin and adipokines as contributors to obesity and the MS: a proposed pathophysiological model of MS centered in irisin, adipokines, and inflammation

**Fig. 4:**
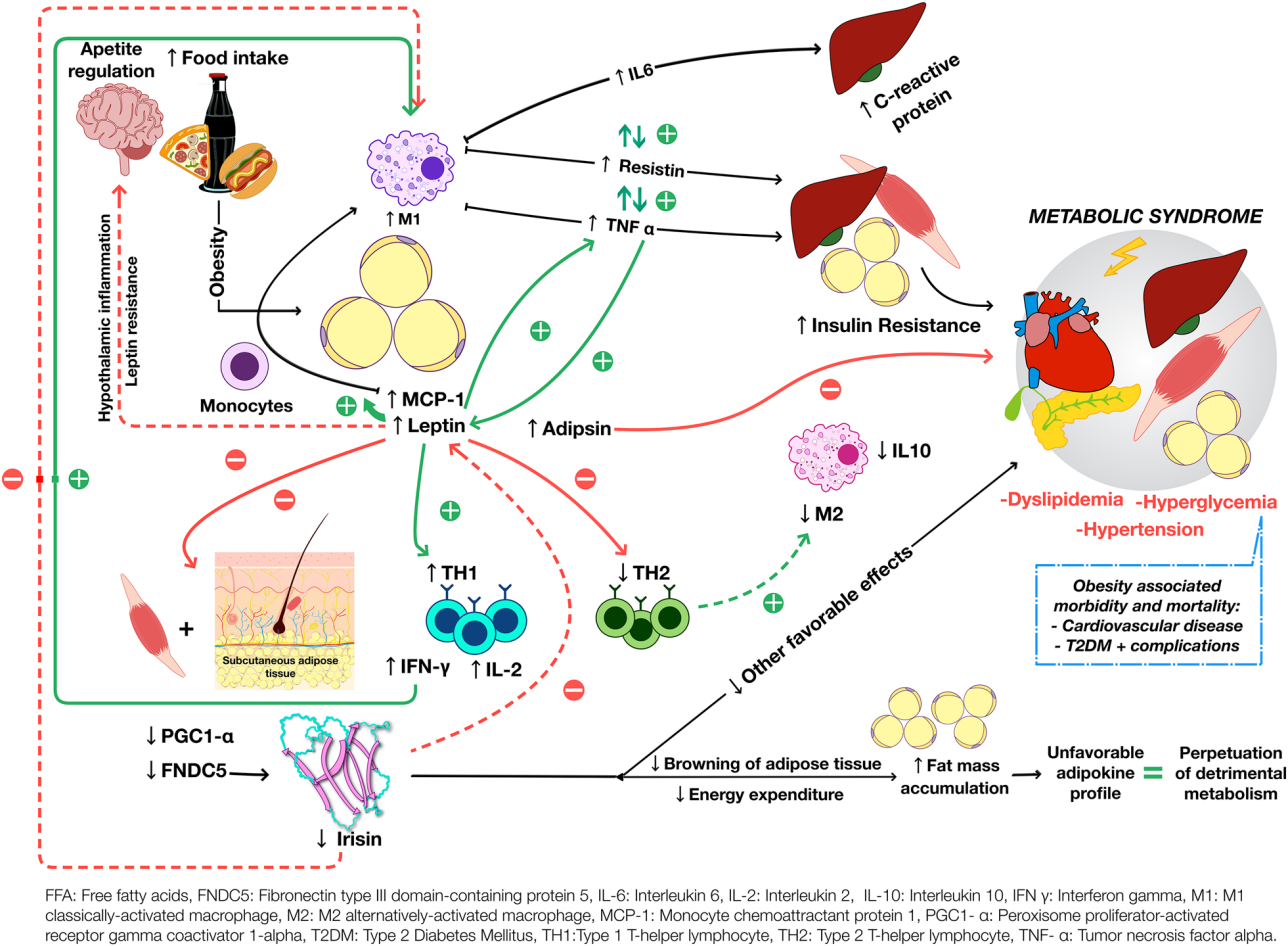
Proposed cross-talk between adipokines and irisin and their contribution to the pathogenesis of MS. Continuous lines indicate an effect that could be observed in context of obesity and MS. Green lines indicate stimulation and red lines indicate inhibition. Dotted red lines indicate a normally present inhibition that may not be present in obesity and MS

Some hypotheses can be drawn regarding the interaction between irisin and adipokines. Figure 4 depicts our view on how the obesity-related profile of adipokines and irisin, with inclusion of cytokines, could contribute to the MS and its complications, as it is well known that obesity and its associated diseases are characterized by a state of chronic low-grade systemic inflammation [91]. In vivo evidence has shown that interactions between classical proinflammatory cytokines and adipokines in obesity are perpetuated through maintenance of positive feedback and suppression of protective negative feedback mechanisms. These ultimately result in insulin resistance and endothelial dysfunction, which give rise to the MS and its complications. In brief, adipocytes from VAT promote M1 classically-activated macrophage infiltration through MCP-1 chemotaxis, which leads to overexpression and secretion of proinflammatory adipocytokines, including resistin, leptin, TNF-α, and IL-6, among others. In turn, autoregulation of adipocytokines occurs through positive and negative feedback mechanisms [7]. For instance, both leptin [92, 93] and resistin [15] upregulate TNF-α and other proinflammatory cytokines, while TNF-α reciprocally upregulates resistin [94] and leptin [95], thereby establishing a detrimental positive feedback loop. Increased levels of leptin accompanied by central leptin resistance in obesity lead to increased food intake, decreased energy expenditure, and further rise in leptin levels. Increased levels of leptin have also been shown to mediate monocyte recruitment and activation [95], and to favor T-helper differentiation to Th_1_ proinflammatory phenotype [96], all of which further enhance the maintenance of inflammation and its metabolic consequences. Considering the previously discussed role of leptin in the regulation of irisin, hyperleptinemia may either increase or decrease the levels of circulating irisin, depending on the relative abundance of distinct adipose tissue types and muscle mass in a given individual, as well as the degree of leptin resistance in such tissues. As our results showed irisin levels to be negatively correlated with the number of cardiometabolic risk factors in the pediatric population, increased energy expenditure through adipose tissue browning induced by irisin may represent a mechanism to counterbalance the metabolic imbalances induced by obesity. Of importance, irisin itself possesses anti-inflammatory properties in vitro [97, 98]. For instance, when murine macrophages were activated with lipopolysaccharide (LPS), the expected proinflammatory activation was found to be blunted following treatment with irisin in a dose-dependent manner, which resulted in decreased expression of the proinflammatory cytokines TNF-α, IL-1β and IL-6 and of monocyte chemoattractant protein-1 (MCP-1) [97]. In adipocytes, irisin has also been found to reduce the expression of TNF-α, IL-6, and leptin, and to increase the expression of the anti-inflammatory and insulin-sensitizing adipokine adiponectin [98]. Hence, low levels of irisin in obese subjects and those with MS may further promote metabolic derangements that are dependent on low-grade systemic inflammation. In brief, obese subjects and those with the MS who have lower levels of irisin, independently of the mechanism involved, may have a blunted compensatory response to adipokine-mediated metabolic imbalances and a perpetuated systemic proinflammatory state that contribute to a continuous deleterious effect cycle.

The study has some limitations. The population studied is from Hispanic ethnicity and thus, our results might not be extrapolated to other populations. Due to the cross-sectional design causality of the relationship of irisin with adipokines cannot be established. Although physical activity was evaluated through a validated questionnaire, we did not measure acute changes of irisin after exercise. Thus, we were unable to observe a cause-effect relationship between irisin and physical activity. Although muscle mass and fat mass in kg were calculated by standardized formulas, slight inaccuracies have to be considered. It is also of importance to highlight concerns raised about the reliability of available irisin ELISA kits due to the lack of standardization among different studies. Hence, results should be cautiously interpreted. However, several strengths are highlighted. This is the first study to establish correlations between circulating irisin and several adipokines in the pediatric population. We also emphasize the possible significance of these associations in obesity from an overall cross-talk perspective, which could offer an insight for further research related to irisin in the pediatric population.

## Conclusions

In summary, this study demonstrated irisin levels to be lower in children with obesity and the MS compared with those with normal weight. Although age and gender did not appear to influence the levels of irisin when considering the entire cohort, significant dispersion in irisin levels was observed among obese girls and girls with the MS, which was not observed in boys. Earlier onset of puberty and accompanying increased fat mass in girls might partly explain this phenomenon. Irisin correlated negatively with anthropometric and metabolic markers. However, after adjusting for BMI% and WC%, no association was observed between irisin and individual biochemical parameters. Noteworthy, lean-fat ratio was a major component influencing levels of irisin. Hence, considering that irisin might have a role as a metabolic regulator, the lower levels of irisin may further exacerbate metabolic imbalances seen in obesity and the MS. Furthermore, irisin correlated negatively with the increased levels of leptin, irisin and adipsin observed in the obese and the MS groups, which may suggest cross-talk between myokines and adipokines in mechanisms leading to the MS. Further mechanistic studies are needed to determine the role of irisin in such cross-talk in the pediatric population. The scarce evidence of the role of irisin in pediatric obesity and MS warrants further studies to corroborate our findings.

## Additional file

**Additional file 1: Table S1.** Comparison of body composition parameters among subgroups divided by gender and age. **Table S2.** Comparison of body composition parameters among children grouped only by age in years. **Table S3.** Laboratory standard values.

## Data Availability

The datasets used and/or analyzed during the current study are available from the corresponding author on reasonable request.

## Abbreviations

BMI: body mass index
DBP: diastolic blood pressure
Fat%: percent body fat
FFA: free fatty acids
FFM: fat-free mass
FM: fat mass
FNDC5: fibronectin type III domain-containing protein 5
HDL-c: high density lipoprotein cholesterol
IFN-γ: interferon gamma
IL-2: interleukin 2
IL-6: interleukin 6
IL-10: interleukin 10
LF ratio: lean-fat ratio
LM: lean mass
M1: classically activated macrophage
M2: alternatively activated macrophage
MCP-1: monocyte chemoattractant protein 1
MS: metabolic syndrome
NCEP-ATP III: National Cholesterol Education Program Adult Treatment Panel III
NW: normal weight
Ob: obese
PAdpw: physical activity in days per week
PAhpd: physical activity in hours per day
PAhpw: physical activity in hours per week
PGC-1α: peroxisome proliferator-activated receptor gamma coactivator 1-alpha
SAT: subcutaneous adipose tissue
SBP: systolic blood pressure
T2DM: type 2 diabetes mellitus
TG: triglycerides
Th_1_: type 1 T-helper lymphocyte
Th_2_: type 2 T-helper lymphocyte
TNF-α: tumor necrosis factor alpha
UCP-1: uncoupling protein 1 (thermogenin)
VAT: visceral adipose tissue
WC: waist circumference
#RF: number of cardiometabolic risk factors
