# Supplementary material for: Myokine–adipokine cross-talk: potential mechanisms for the association between plasma irisin and adipokines and cardiometabolic risk factors in Mexican children with obesity and the metabolic syndrome
Source: Diabetol Metab Syndr. 2019 Aug 5;11:63. doi: 10.1186/s13098-019-0458-2 (PMC6683550; doi:10.1186/s13098-019-0458-2)
Supplement: Supplementary file 1 — Additional file 1: Table S1. Comparison of body composition parameters among subgroups divided by gender and age. Table S2. Comparison of body composition parameters among children grouped only by age in years. Table S3. Laboratory standard values. [file 13098_2019_458_MOESM1_ESM.docx]

| **Additional file 1: Table S1. Comparison of body composition parameters among subgroups divided by gender and age** | | | | | |
| --- | --- | --- | --- | --- | --- |
|  | **Normal weight** (n = 46) | | | |  |
|  | **Males** (n = 23) | | **Females** (n = 23) | |  |
|  | **6-9 years old** (n = 11) | **10-12 years old** (n = 12) | **6-9 years old** (n = 18) | **10-12 years old** (n = 5) |  |
| Body fat percent (%) | 13.3 [12.9-15.8] | 13.10 [11.95-16.40] | 14.70 [12.80-17.20] | 18.20 [14.40-18.40] |  |
| Fat mass (kg) | 3.34 [2.72-3.62] | 4.11 [3.18-5.48] | 3.80 [3.04-4.93] | 6.66 [5.26-7.52] |  |
| Lean mass (kg) | 5.61 [4.53-7.55] | 7.17 [6.07-8.40] | 6.21 [5.39-6.68] | 9.10 [7.93-9.11] |  |
| Fat-free mass (kg) | 21.25 [17.16-23.66]^C,D^ | 26.84 [23.71-28.64]^C,D^ | 20.46 [18.97-23.07]^A,B^ | 29.54 [28.04-31.24]^A,B^ |  |
| Lean-fat ratio | 1.74 [1.26-2.11] | 1.72 [1.10-2.02] | 1.64 [1.31-1.98] | 1.37 [1.17-1.75] |  |
|  | **Obese** (n = 40) | | | |  |
|  | **Males** (n = 20) | | **Females** (n = 20) | |  |
|  | **6-9 years old** (n = 12) | **10-12 years old** (n = 8) | **6-9 years old** (n = 16) | **10-12 years old** (n = 4) |  |
| Body fat percent (%) | 31.85 [31.20-34.60] | 33.65 [32.30-36.80] | 32.6 [29.45-38.40] | 35.5 [32.65-39.25] |  |
| Fat mass (kg) | 12.89 [11.40-13.38]^C^ | 16.50 [15.27-18.13] | 11.85 [8.85-15.46]^A^ | 18.06 [15.10-22.74] |  |
| Lean mass (kg) | 6.56 [4.71-7.53] | 5.87 [5.29-6.86] | 6.77 [6.37-7.36] | 7.70 [6.45-9.86] |  |
| Fat-free mass (kg) | 26.76 [24.61-28.20]^C^ | 32.39 [30.93-34.15]^C^ | 23.78 [21.85-24.86]^A,B^ | 32.78 [30.57-35.64] |  |
| Lean-fat ratio | 0.43 [0.39-0.63] | 0.37 [0.33-0.40] | 0.57 [0.43-0.71] | 0.41 [0.32-0.61] |  |
|  | **Metabolic syndrome** (n = 40) | | | |  |
|  | **Males** (n = 20) | | **Females** (n = 20) | |  |
|  | **6-9 years old** (n = 10) | **10-12 years old** (n = 10) | **6-9 years old** (n = 12) | **10-12 years old** (n = 8) |  |
| Body fat percent (%) | 34.95 [32.40-48.90] | 38 [32.20-53.90] | 36.85 [32.30-39.60] | 39.00 [35.95-40.75] |  |
| Fat mass (kg) | 15.52 [13.80-23.60] | 22.99 [16.33-29.54] | 14.26 [11.22-19.26] | 18.84 [15.96-27.28] |  |
| Lean mass (kg) | 8.20 [6.74-9.56]] | 7.79 [5.71-8.97] | 6.97 [5.92-7.93] | 7.81 [7.15-9.81] |  |
| Fat-free mass (kg) | 26.75 [23.64-28.80]^C^ | 33.30 [30.38-37.04] | 24.32 [22.35-30.69]^A^ | 31.67 [28.74-38.52] |  |
| Lean-fat ratio | 0.45 [0.34-0.65] | 0.43 [0.17-0.45] | 0.53 [0.36-0.70] | 0.43 [0.36-0.53] |  |
| Data are presented as median and interquartile range*. p*<0.05 versus (A) Males 6-9 years old, (B) Males 10-12 years old, (C) Females 6-9 years old, and (D) Females 10-12 years old in their respective group (Normal weight, obese, or metabolic syndrome) with non-parametric multiple comparisons test. | | | | | |

| **Additional file 1: Table S2. Comparison of body composition parameters among children grouped only by age in years** | | | | | | | |  | |
| --- | --- | --- | --- | --- | --- | --- | --- | --- | --- |
|  | **6** (n = 10) | **7** (n = 24) | **8** (n = 25) | **9** (n = 20) | **10** (n = 24) | **11** (n = 15) | **12** (n = 8) | |  |
| Body fat percent (%) | 21.00 [14.20-34.90] | 29.05 [14.15-34.75] | 31.40 [17.20-35.90] | 30.00 [17.80-36.15] | 31.90 [13.10-37.10] | 33.60 [18.90-39.00] | 32.40 [23.85-38.85] | |  |
| Fat mass (kg) | 5.25 [2.69-11.91] | 9.00 [3.40-14.04] | 10.90 [4.14-13.95] | 12.03 [5.37-17.02] | 14.36 [4.11-16.57] | 17.67 [6.27-24.22] | 17.44 [9.36-24.81] | |  |
| Lean mass (kg) | 5.34 [4.84-5.58]^D,G^ | 6.44 [5.53-7.19] | 6.40 [5.72-7.49] | 7.54 [6.58-8.49]^A^ | 7.17 [5.98-8.56] | 6.72 [5.81-9.48] | 8.08 [7.37-9.35]^A^ | |  |
| Fat-free mass (kg) | 20.35 [17.28-23.91]^D,E,F,G^ | 22.45 [19.71-23.94]^D,E,F,G^ | 23.66 [21.32-24.89]^E,F,G^ | 27.52 [24.09-30.51]^A,B^ | 28.24 [26.62-31.10]^A,B,C^ | 31.72 [30.13-35.58]^A,B,C^ | 35.41 [32.93-38.89]^A,B,C^ | |  |
| Lean-fat ratio | 0.90 [0.41-1.78] | 0.67 [0.47-1.54] | 0.61 [0.43-1.47] | 0.71 [0.49-1.19] | 0.51 [0.37-1.72] | 0.45 [0.41-1.08] | 0.46 [0.34-0.81] | |  |
| Data are presented as median and interquartile range*. p*<0.05 versus (A) 6 years old, (B) 7 years old, (C) 8 years old, (D) 9 years old, (E) 10 years old, (F) 11 years old, and (G) 12 years old with non-parametric multiple comparisons test. | | | | | | | | | |

| **Additional file 1: Table S3. Laboratory Standard Values** | | |  |
| --- | --- | --- | --- |
|  | **Normal Values** | **Units** |  |
| Glucose | 60-100 | mg/dL |  |
| Triglycerides | ≤110 | mg/dL |  |
| HDL-cholesterol | ≥40 | mg/dL |  |
| Standard values from laboratory are based on the ADA and the NCEP-III criteria | | | |
